# Draft Genome Sequences of Clinical Isolates of Serotype 6E *Streptococcus pneumoniae* from Five Asian Countries

**DOI:** 10.1128/genomeA.01728-16

**Published:** 2017-03-09

**Authors:** In Ho Park, Jin Yang Baek, Jae-Hoon Song, Kwan Soo Ko, Kyung-Hyo Kim

**Affiliations:** a Center for Vaccine Evaluation and Study, Medical Research Institute, Ewha Womans University, Seoul, Republic of Korea; b Department of Pediatrics, School of Medicine, Ewha Womans University, Seoul, Republic of Korea; c Asia Pacific Foundation for Infectious Diseases (APFID), Seoul, Republic of Korea; d Division of Infectious Diseases, Samsung Medical Center, Sungkyunkwan University School of Medicine, Seoul, Republic of Korea; e Department of Molecular Cell Biology, Sungkyunkwan University School of Medicine, Suwon, Republic of Korea

## Abstract

Although serotype 6E *Streptococcus pneumoniae* consistently expresses capsules of either vaccine-serotype 6A or 6B, certain genetic variants of serotype 6E may evade vaccine induced immunity. Thus, draft genome sequences from five clinical isolates of serotype 6E from each of five different Asian countries have been generated to provide insight into the genomic diversity in serotype 6E strains.

## GENOME ANNOUNCEMENT

*Streptococcus pneumoniae* infections remain a leading cause of morbidity and mortality worldwide. A seven-valent pneumococcal conjugate vaccine, PCV7, introduced first in the United States in 2000, has been introduced in six Asian countries (South Korea, Taiwan, Thailand, Philippines, Saudi Arabia, and Japan) between 2003 and 2010 (1). PCV7 has been improved to a 13-valent pneumococcal conjugate vaccine (including both serotypes 6A and 6B) as of 2010 for more serotype coverage. However, the incidence of carriage by some nonvaccine serotypes of pneumococci is still increasing worldwide (2–5).

Generally, serogroup 6 includes two serotypes with vaccine protection (6A and 6B) as well as two others (6C and 6 D) (6, 7), but recent studies have described four additional serotypes, 6E, 6F, 6G, and 6H. Serotype 6E has two different repeating units from among the four previously known serotypes, 6A, 6B, 6C, and 6D (8, 9), differentiating it from the three bivalent serotypes 6F, 6G, and 6H. Serotype 6E also has great genetic variation in the capsule biosynthesis gene (*cps*) locus which is strikingly different from the *cps* loci of known serogroup 6 members (10). Serotype 6E can produce capsule polysaccharides of either serotype 6A or 6B (unpublished data). A previous study revealed that most serotype 6E isolates from Asian countries belong to clonal complex (CC) 90, which is highly antimicrobial resistant (11).

Herein, we report the genome sequences of five serotype 6E strains collected from five different Asian countries, including strains HK02-14, J01-5, K13-21, M12-6, and V03-9 (10–13). Genomic DNA sequencing was performed using an Illumina MiSeq platform, and the draft *de novo* genome assembly was generated using CLC Genomics Workbench version 5.5.2 software.

An average of 20.47 million paired-end reads per strain were collected, and the number of contigs, the contig *N*_50_ length, and the total sequence length for each strain were derived as follows: 251, 24.7 kb, and 2.18 Mb for HK02-14; 323, 24.6 kb, and 2.17 Mb for J01-5; 1,754, 35.6 kb, and 2.74 Mb for K13-21; 673, 35.5 kb, and 2.29 Mb for M12-6; and 266, 22.9 kb, and 2.15 Mb for V03-9, respectively.

Open reading frames were identified and annotated with the NCBI Prokaryotic Genome Automatic Annotation Pipeline (14). The number of putative protein-coding genes and tRNAs in each strain were as follows: 2,037 and 17 for HK02-14; 2,033 and 26 for J01-5; 2,132 and 27 for K13-21; 2,070 and 32 for M12-6; and 2,029 and 14 for V03-9.

This genome sequence information represents four serotype 6E strains in CC90 and one singleton strain (ST1404). A previous study reported that incomplete coverage of serotype 6E by PCV7 may be due to the clonal dissemination of a few CCs, particularly CC90 (11). These whole-genome sequences may be helpful in verifying whether serotype 6E, which is prevalent worldwide, escapes the vaccine and shows high antimicrobial resistance.

### Accession number(s).

The GenBank accession numbers for the first versions of these sequences are LOEA00000000, LOEB00000000, LODY00000000, LODX00000000, and LODZ00000000 for strains HK02-14, J01-5, K13-21, M12-6, and V03-9, respectively.
